# Advantageous characteristics of the diatom *Chaetoceros gracilis* as a sustainable biofuel producer

**DOI:** 10.1186/s13068-016-0649-0

**Published:** 2016-11-03

**Authors:** Hiromi Tokushima, Natsuko Inoue-Kashino, Yukine Nakazato, Atsunori Masuda, Kentaro Ifuku, Yasuhiro Kashino

**Affiliations:** 1 Graduate School of Life Science, University of Hyogo, 3-2-1 Koto, Kamigori, Ako-gun, Hyogo 678-1297 Japan; 2 Yanmar Environmental Sustainability Support Association, Umeda Gate-Tower, 1-9 Tsuruno, Kita-ku, Osaka, 530-8311 Japan; 3 College of Agriculture, Tamagawa University, 6-1-1 Tamagawagakuen, Machida, Tokyo, 194-8610 Japan; 4 Graduate School of Biostudies, Kyoto University, Kyoto, 606-8502 Japan

**Keywords:** *Chaetoceros gracilis*, Diatom, Biofuel, Lipid, Triacylglycerols, Fucoxanthin, EPA, Chlorophyll

## Abstract

**Background:**

Diatoms have attracted interest as biofuel producers. Here, the contents of lipids and photosynthetic pigments were analyzed in a marine centric diatom, *Chaetoceros gracilis*. This diatom can be genetically engineered using our previously reported transformation technique and has a potential to produce valuable materials photosynthetically. Sustainable culture conditions for cost-effective production of biological materials under autotrophic conditions with atmospheric carbon dioxide were investigated in the laboratory. A large-scale, open-air culture was also performed.

**Results:**

Cell population doubling time was ~10 h under continuous illumination without CO_2_ enrichment, and large amounts of triacylglycerols (TAG) and fucoxanthin accumulated under a wide range of salinity and nutrient conditions, reaching ~200 and 18.5 mg/L, respectively. It was also shown that *C. gracilis* produced high amounts of TAG without the need for nitrogen or silica deprivation, which is frequently imposed to induce lipid production in many other microalgae. Furthermore, *C. gracilis* was confirmed to be highly tolerant to changes in environmental conditions, such as salinity. The diatom grew well and produced abundant lipids when using sewage water or liquid fertilizer derived from cattle feces without augmented carbon dioxide. High growth rates (doubling time <20 h) were obtained in a large-scale, open-air culture, in which light irradiance and temperature fluctuated and were largely different from laboratory conditions.

**Conclusions:**

The ability of this microalga to accumulate TAG without nutrient deprivation, which incurs added labor, high costs, and complicates scalability, is important for low-cost industrial applications. Furthermore, its high tolerance to changes in environmental conditions and high growth rates observed in large-scale, open-air culture implied scalability of this diatom for industrial applications. Therefore, *C. gracilis* would have great potential as a biofactory.

## Background

Diatoms emerged on our planet ~150 million years ago [1, 2], and they are considered one of the major sources of crude oil [3–5]. Currently, diatoms are responsible for nearly one-quarter of the annual global photosynthetic production of organic matter, which is nearly equal to the proportion attributed to tropical rain forests [6, 7]. In many diatomaceous species, assimilated carbon is accumulated in cells as triacylglycerol (TAG) that accounts for 25–45% of their cell dry weight [8, 9], while their silica shell makes up 40–78% of their weight [10]. In addition, diatoms contain many types of fatty acids (FAs) and related organic molecules [11]. As a result, diatoms have attracted interest as biofuel producers. Currently, among the 20,000–200,000 species of diatoms, only a few have been studied as potential biofuel producers [12–14], including *Phaeodactylum tricornutum*, *Thalassiosira pseudonana*, *Thalassiosira weissflogii*, *Cyclotella cryptica* [9], *Fistulifera solaris* [15], *Chaetoceros gracilis* (*C. muelleri*) [16, 17], *Cylindrotheca* spp., and *Nitzschia* spp. [8]. In laboratory-scale experiments, nitrogen or silica deprivation is frequently used to induce lipid production and accumulation [8, 17–23].

In addition to organic precursors for biofuels, valuable metabolites, such as eicosapentaenoic acid (EPA) and docosahexaenoic acid (DHA) [24], as well as photosynthetic pigments, such as fucoxanthin, diadinoxanthin, diatoxanthin, and chlorophylls (Chl) *a* and *c* [25–27], are found in diatoms. EPA, DHA, and fucoxanthin are also clinically important [24, 28–33]. Fucoxanthin is a major pigment in diatoms and some types of haptophytes [34–36] as well as brown algae [37], and its function is to harvest light energy by associating with fucoxanthin-chlorophyll binding protein [38]. Fucoxanthin’s inhibitory effects on cancer cells [28–33] and its antioxidant effects [37] have attracted attention regarding its potential use as a potent drug and dietary supplement similar to astaxanthin [39]. Chl *c* is a photosynthetic pigment possessed by brown-colored photosynthetic organisms, such as diatoms and brown algae. In contrast to regular Chls that belong to the chlorin family of compounds, C17 and C18 in pyrrole ring D of Chl *c* are connected by a double bond. Therefore, Chl *c* belongs to the porphyrin family of compounds [40]. Until now, three types of Chl *c* are known, namely Chl *c*_1_, *c*_2_, and *c*_3_ [41–43]. Among them, Chl *c*_1_ and *c*_2_ are found in most diatoms. Chl *c*_3_ is a haptophyte marker pigment [36] and possessed by some diatoms, such as *Nitzschia bilobata* [42]. An esterified form of Chl *c* is also known in haptophytes [44–47]. Diadinoxanthin and diatoxanthin are the pigments involved in the diadinoxanthin cycle [34, 48–53], a xanthophyll cycle variant in diatoms [54]. Diadinoxanthin is converted into diatoxanthin under high light conditions to protect photosystems through nonphotochemical quenching. When grown under high light, the diadinoxanthin cycle pigment content usually increases [34, 48–53].

It is desirable to raise the yield of these valuable metabolites through efficient diatom photosynthesis and growth. In this context, genetic manipulation techniques [55–61] are crucial for converting diatoms into “biofactories,” and it has been demonstrated that metabolic pathways can be modified using such techniques [62]. For example, knockdown of a multifunctional lipase/phospholipase/acyltransferase causes increased lipid yields in the marine centric diatom *T. pseudonana* [63]. In *P. tricornutum*, malic enzyme overexpression enhances total lipid production up to 2.5-fold without sacrificing growth rate [64]. The transformation efficiency of *P. tricornutum* was considerably improved in our recent work [65]. Genomic analyses of *T. pseudonana* [66], *P. tricornutum* [67], and the oleaginous diatom *F. solaris* [15] have supported the advancement of diatom genetic manipulation, and will facilitate industrial applications of these organisms.

Diatoms are attracting increasing attention as biofactories for the production of many kinds of metabolites. However, large-scale cultivation—a prerequisite for industrial application—has not been successful for most phytoplankton, including diatoms. The limited number of successful examples includes *Chlorella*, *Hematococcus*, *Euglena*, and *Spirulina* species, which are mainly used for production of dietary supplements or cosmetic ingredients. Although successful large-scale cultivation of these species is advantageous for industrial production, there are no established techniques for genetic transformation of these microalgae.

In nature, diatoms sometimes grow in dense accumulations called “spring blooms” [26, 68], which can be detected by satellite [69]. Thus, large-scale diatom cultivation at a high density is feasible. Currently, large-scale diatom cultivation is only performed for feed production, such as for shrimp larviculture in southeastern Asia [70] and clam larviculture in Japan [71]. Among thousands of diatom species, the marine centric diatom *C. gracilis* is considered an ideal species for shrimp larviculture owing to its nutritional content and size as well as its ability to be cultivated on an industrial scale [70]. There is also existing knowledge of its physiological characteristics regarding photosynthesis and oil production [34, 38, 72, 73]. Furthermore, we recently established a stable and efficient genetic transformation technique for *C. gracilis* [74]. Therefore, *C. gracilis* appears to be a good candidate for balancing high growth rate with high metabolite yield. In this work, *C. gracilis*’ growth rate and its yield of several metabolites were investigated to demonstrate that *C. gracilis* is a promising species as a biofactory to produce beneficial metabolites, including potential drug candidates. The effects of changes in environmental conditions, such as nutrient availability, were also assessed in terms of facilitating the production of these materials at a reasonable cost.

## Results

### Effects of vitamins on growth

**Fig. 1:**
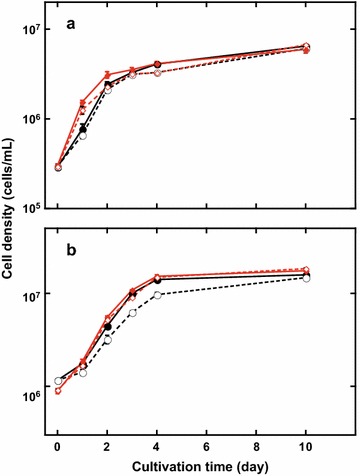
Growth of *C. gracilis* (**a**) and *P. tricornutum* (**b**) with or without vitamins. Cells precultured in same culture medium at least twice, then inoculated into fresh medium at logarithmic or stationary phase (*red* or *black lines*, respectively) at an OD_730_ of ~0.07; cells cultivated at room temperature with photon irradiance of 50 µmol photons m^−2^ s^−1^ in F/2-enriched seawater with (*closed symbols* and *solid lines*) or without (*open symbols* and *dashed lines*) vitamins; and ambient air bubbled through media

F/2 medium and Daigo IMK medium, which differs from F/2 medium mainly in nitrate concentration (200 vs. 75 ppm in F/2), contain vitamins, including vitamin B_12_. The effects of vitamin deprivation on *C. gracilis* growth were assessed using F/2-enriched seawater made with sea salts (hereafter “seawater”). The possible effects of preculture history, with or without vitamins, were excluded by subculturing cells at least twice in the same medium with or without vitamins; each subculture was incubated for ~1 week. Cells in vitamin-deficient medium (Fig. 1a, open symbols) grew at almost the same doubling time (DT), 12.0 h, as cells in vitamin-containing medium (Fig. 1a, closed symbols; DT, 10.7 h). When cells were subcultured during stationary phase, growth rate was somewhat lower than when subcultured during logarithmic phase (Fig. 1a; Table 1). Cell density and triacylglycerols (TAG) produced after 10 days of culture were not affected by eliminating vitamins from the culture medium (Table 1).

**Table 1:** Effects of vitamins on growth and TAG content of two diatoms, *C. gracilis* and *P. tricornutum*

	Inoculation at logarithmic phase		Inoculation at stationary phase	
	+ vitamins	− vitamins	+ vitamins	− vitamins
*C. gracilis*				
Doubling time (h)	10.7 ± 0.6	12.0 ± 0.6	12.6 ± 0.6	12.5 ± 0.3
Cell density on 4th day (10^6^ cells/mL)	4.14 ± 0.22	3.26 ± 0.07	4.08 ± 0.15	3.30 ± 0.07
Cell density on 10th day (10^6^ cells/mL)	6.28 ± 0.25	6.68 ± 0.04	6.48 ± 0.27	6.08 ± 0.19
Volume-specific TAG on 10th day (mg/L)	232 ± 9	190 ± 5	216 ± 9	209 ± 3
Cell-specific TAG on 10th day (pg/cell)	38.5 ± 1.1	29.0 ± 0.2	33.3 ± 0.1	34.4 ± 0.8
*P. tricornutum*				
Doubling time (h)	14.5 ± 0.1	14.4 ± 0.2	17.5 ± 0.8	20.1 ± 0.9
Cell density on 4th day (10^6^ cells/mL)	15.3 ± 0.6	14.9 ± 0.2	14.1 ± 0.2	9.68 ± 0.52
Cell density on 10th day (10^6^ cells/mL)	17.5 ± 0.3	18.4 ± 0.3	15.8 ± 0.2	14.7 ± 0.6
Volume-specific TAG on 10th day (mg/L)	179 ± 4	181 ± 5	152 ± 6	54.5 ± 5.4
Cell-specific TAG on 10th day (pg/cell)	10.2 ± 0.3)	9.84 ± 0.20)	9.92 ± 0.67	3.69 ± 0.20

Cells cultivated in F/2-enriched seawater with or without vitamins (B_12_, biotin, and thiamine-HCl), and *n* = 3–4

The same experiment was performed with the model diatom *P. tricornutum* (Fig. 1b; Table 1). This diatom’s growth rate was lower than that of *C. gracilis* in F/2-enriched seawater with vitamins (DT, 14.5 vs. 10.7 h, respectively). When *P. tricornutum* cells were subcultured during logarithmic phase, growth rate and TAG production were not affected by eliminating vitamins. However, when cells were subcultured during stationary phase without vitamins, growth rate and TAG production were reduced. Note that, when cells were subcultured at stationary phase, cell density was much higher for *P. tricornutum* than for *C. gracilis*, but the total TAG was much lower in *P. tricornutum* than in *C. gracilis*.

### Effects of salinity on growth

*Chaetoceros gracilis* cells were cultivated in different seawater dilutions. Sea salts were usually used at a concentration of 4% (w/v). When sea salt concentrations were reduced to 2 or 3% (w/v), growth rate, cell density at the 4th and 10th days of cultivation, and TAG content of *C. gracilis* were not affected (Table 2). When *C. gracilis* was grown in 1% sea salt medium, growth rate was slightly reduced (DT, 12.4 h) but TAG production notably increased (Table 2).

**Table 2:** Effects of diluting seawater on growth and TAG content of *C. gracilis*

	Concentration of sea salts (%)			
	1	2	3	4
Doubling time (h)	12.4 ± 3.1	10.0 ± 0.6	10.2 ± 0.2	10.5 ± 0.8
Cell density on 4th day (10^6^ cells/mL)	4.73 ± 0.23	5.66 ± 0.20	6.09 ± 0.27	6.16 ± 0.24
Cell density on 10th day (10^6^ cells/mL)	6.34 ± 0.25	6.35 ± 0.33	7.24 ± 0.28	6.40 ± 0.65
Volume-specific TAG on 10th day (mg/L)	227 ± 14	194 ± 16	196 ± 26	189 ± 23
Cell-specific TAG on 10th day (pg/cell)	35.4 ± 1.1	35.0 ± 3.0	30.7 ± 3.0	33.2 ± 3.2

Cells cultivated in various concentrations of seawater made from sea salts, seawater enriched with F/2 medium without vitamins, and *n* = 3–4

Because small decreases in sea salt concentration did not appear to affect *C. gracilis* growth, the effects of salinity on growth were assessed. Base culture media were prepared by varying the NaCl concentration and supplementing with MgSO_4_, CaCl_2_, KCl, and NaHCO_3_ to obtain concentrations roughly equivalent to those in seawater. With salinity similar to the 3% NaCl (w/v) in natural seawater, *C. gracilis* growth characteristics (Table 3) were similar to those in normal culture medium composed of sea salts and F/2 (Fig. 1; Table 1). When the NaCl concentration was increased from 3 to 4%, growth rate was decreased and TAG production reduced to less than half the previous rates. Conversely, as described above, when *C. gracilis* was grown at NaCl concentrations below 3%, growth characteristics improved. The highest growth rate, cell density, and TAG production were observed when cells were grown in 1% NaCl medium. Interestingly, these cells grew well even without NaCl; growth rate and TAG production were higher with 0% NaCl than with 4%, even though *C. gracilis* is a marine diatom. However, when *C. gracilis* cells grown in 3% NaCl medium were transferred to 0% NaCl conditions, a large proportion of these cells did not survive and the remaining cells began to proliferate after about 1 day.

**Table 3:** Effects of salinity on growth and TAG content of two diatoms, *C. gracilis* and *P. tricornutum*

	Concentration of NaCl (%)					
	0	0.5	1	2	3	4
*C. gracilis*						
Doubling time (h)	11.1 ± 0.5	9.52 ± 0.48	9.16 ± 0.17	9.75 ± 0.20	11.3 ± 1.5	15.7 ± 0.4
Cell density on 4th day (10^6^ cells/mL)	2.41 ± 0.19	4.41 ± 0.67	5.32 ± 0.14	5.24 ± 0.26	3.64 ± 0.80	2.24 ± 0.81
Cell density on 10th day (10^6^ cells/mL)^a^	3.89 ± 0.45	5.30 ± 0.15	6.29 ± 0.31	6.47 ± 0.43	3.63 ± 0.32	2.71 ± 0.23
Volume-specific TAG on 10th day (mg/L)	223 ± 18	234 ± 43	272 ± 4	223 ± 6	172 ± 3	69.3 ± 28.6
Cell-specific TAG on 10th day (pg/cell)^a^	73.1 ± 7.3	46.2 ± 4.9	47.0 ± 2.5	35.5 ± 3.9	25.5 ± 0.6	19.3 ± 5.0
*P. tricornutum*						
Doubling time (h)	n.d.	n.d.	14.6 ± 0.2	15.8 ± 0.4	15.4 ± 0.7	19.0 ± 0.9
Cell density on 4th day (10^6^ cells/mL)	n.d.	n.d.	14.9 ± 0.4	15.4 ± 0.2	13.2 ± 0.6	11.6 ± 0.3
Cell density on 10th day (10^6^ cells/mL)	n.d.	n.d.	16.4 ± 1.0	16.6 ± 9.2	14.9 ± 0.1	13.8 ± 0.6
Volume-specific TAG on 10th day (mg/L)	n.d.	n.d.	183 ± 11	180 ± 9	158 ± 14	161 ± 15
Cell-specific TAG on 10th day (pg/cell)	n.d.	n.d.	11.2 ± 0.4	10.9 ± 1.1	10.6 ± 0.8	11.7 ± 0.6

Cells cultivated in media containing various NaCl concentrations; media enriched with F/2 without vitamins and MgSO_4_, CaCl_2_, KCl, and NaHCO_3_ added, as described in “Methods” section

*n.d.* not determined; *n* = 3–4

^a^ *n* = 2

Growth characteristics at various salinities were different for *P. tricornutum* compared with *C. gracilis* (Table 3). Growth rate and total TAG content of *P. tricornutum* were relatively constant despite changes in NaCl concentration from 1 to 4%. These results were generally inferior to those of *C. gracilis*.

### Use of urea instead of nitrate as a nitrogen source

In general, nitrate is the most expensive nutrient for phytoplankton culture and thus the effects of substituting urea for nitrate were assessed (Table 4). In F/2-enriched seawater, *C. gracilis* grew much faster with urea than nitrate (DTs, 8.9 and 10.3 h, respectively), and TAG production and cell density were higher with urea than nitrate. When cells were grown with urea in 1% NaCl-based medium, no effects on growth or TAG production were observed. When 70 ppm urea was present in the medium along with nitrate, growth and TAG accumulation were slightly suppressed. When 70 ppm urea was added to artificial seawater (Marine Art SF-1) enriched with Daigo IMK medium, growth was accelerated, but TAG production was slightly reduced.

**Table 4:** Effects of urea on growth and TAG content of *C. gracilis*

	Seawater			Marine Art		1% NaCl-based media		
	F/2	Urea-F/2	F/2 + 70 ppm urea	IMK	IMK + 70 ppm urea	F/2	Urea-F/2	F/2 + 70 ppm urea
Doubling time (h)	10.3 ± 0.1	8.93 ± 0.10	10.5 ± 0.1	10.2 ± 0.4	8.61 ± 0.25	9.16 ± 0.17	9.28 ± 0.16	9.63 ± 0.40
Cell density on 4th day (10^6^ cells/mL)	4.72 ± 0.05	6.01 ± 0.16	6.36 ± 0.06	5.22 ± 0.22	5.67 ± 0.02	5.37 ± 0.37	5.36 ± 0.13	5.52 ± 0.10
Cell density on 10th day (10^6^ cells/mL)	6.64 ± 0.01	7.04 ± 0.02	8.31 ± 0.18	6.73 ± 0.14	6.19 ± 0.05	6.59 ± 0.34	5.51 ± 0.22	7.08 ± 0.13
Volume-specific TAG on 10th day (mg/L)	163 ± 3	179 ± 9	150 ± 6	87.6 ± 5.3	76.6 ± 5.0	272 ± 4	260 ± 6	159 ± 15
Cell-specific TAG on 10th day (pg/cell)	24.1 ± 0.8	26.7 ± 2.6	18.0 ± 0.5	13.8 ± 1.1	11.9 ± 0.9	25.7 ± 1.9	47.4 ± 2.4	21.1 ± 2.1

Cells cultivated in media enriched with F/2 without vitamins; urea-F/2 medium contained urea not nitrate at same molar concentration; F/2 + 70 ppm urea and IMK + 70 ppm urea media contained urea in addition to nitrate; 1% NaCl-based medium in place of seawater contained 1% NaCl and MgSO_4_, CaCl_2_, KCl, and NaHCO_3_ added, as described in “Methods” section; and *n* = 3

### Use of sewage water as a nutrient source

**Fig. 2:**
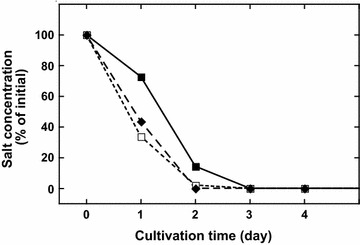
Changes in concentrations of three nutrient salts during the course of diatom growth. *C. gracilis* grown in F/2-enriched 1% NaCl medium without vitamins, and concentrations of nitrate (*closed boxes*), silicate (*closed diamonds*), and phosphate (*open boxes*) determined at indicated times after cell removal by centrifugation; values normalized at day 0; initial concentrations were 50.0, 16.4, and 2.26 ppm for nitrate, silicate, and phosphate, respectively; and some *error bars* smaller than symbols (*n* = 3)

Because *C. gracilis* can grow well in various salinities, cells were grown in artificial seawater diluted with sewage water (Table 5). When cells grown in F/2-enriched artificial seawater without vitamins were subcultured (1st subculture) in seawater diluted with the same volume of sewage water (50% by vol), growth and TAG production were slightly reduced compared with values observed with F/2-enriched artificial seawater. When seawater diluted with two volumes of sewage water (67% by vol) was used, growth rate of the 1st subculture was further reduced (DT, 12.9 and 15.4 h in 50 and 67% sewage water, respectively), but total TAG remained almost the same. Water quality analysis showed that the phosphate concentration in sewage water obtained from Harima Kogen Higashi Waterworks was comparable to that in F/2 and Daigo IMK media (Table 6). The nitrate concentration was much lower in sewage water than in F/2 and Daigo IMK media, and similar values are found in urban sewage treatment plants. Nitrate was a relatively rich nitrogen source for diatom growth in F/2 and Daigo IMK media. In this case, phosphate and silicon were consumed faster than nitrate when cells were grown in F/2 medium (Fig. 2), resulting in lack of growth after day 4 (Fig. 1). This might have been the reason why *C. gracilis* grew and accumulated TAG in sewage water, despite sewage water’s apparently poor nutrient content. However, when cells were subcultured again (2nd subculture, Table 5) in the same sewage water culture medium, subsequent growth was reduced, which might have resulted from low phosphate content in sewage water culture media made by mixing sewage water with seawater containing no phosphate. The final phosphate concentration should be 2.0 and 2.7 ppm in 50 and 67% sewage water culture media, respectively, which was lower than 3.46 ppm in F/2-enriched culture medium (Table 6). Although nitrate concentration was also low in sewage water (Table 6), ammonium should have served as a nitrogen source [75–77]. Ammonium concentrations in 50 and 67% sewage water culture media were 9.0 and 12 ppm, respectively. Because the molecular weight of ammonium is 3.5 times less than nitrate, these concentrations are equivalent to 31 and 42 ppm of nitrate, respectively, which was somewhat lower than that in F/2 medium. As nitrate was consumed at a slower rate than phosphate (Fig. 2), the effect of a somewhat lower nitrogen concentration in sewage water culture media on growth and production might be limited.

**Table 5:** Use of sewage water as the nutrient source in culture media

	50% sewage water		67% sewage water	
	1st subculture	2nd subculture	1st subculture	2nd subculture
Doubling time (h)	12.9 ± 0.4	19.2 ± 0.5	15.4 ± 0.5	18.3 ± 0.1
Cell density on 4th day (10^6^ cells/mL)	5.06 ± 0.02	4.24 ± 0.04	5.29 ± 0.39	4.79 ± 0.05
Cell density on 10th day (10^6^ cells/mL)	6.17 ± 0.08	4.95 ± 0.01	6.19 ± 0.14	5.99 ± 0.05
Volume-specific TAG on 10th day (mg/L)	156 ± 4	135 ± 9	203 ± 9	164 ± 7
Cell-specific TAG on 10th day (pg/cell)	25.2 ± 0.6	27.3 ± 1.8	32.8 ± 1.4	27.4 ± 1.0

*Chaetoceros gracilis* cultured in seawater mixed with one or two volumes of sewage water (50 and 67% sewage water), cells used to inoculate sewage water media cultivated first in F/2-enriched seawater (1st subculture) or subcultured once in same sewage water medium (2nd subculture), and *n* = 3

**Table 6:** Concentrations of four nutrient salts in sewage water and liquid fertilizer

	F/2 (ppm)	Daigo IMK (ppm)	Sewage water (ppm)	Liquid fertilizer (ppm)
Phosphate	3.46	3.66	4	200
Nitrate	54.7	146	(7)	0
Nitrite	0	0	0.5	0
Ammonium	0	0.92	18	3000

Seawater prepared with sea salts enriched with F/2 medium and with Marine Art SF-1 enriched with Daigo IMK medium and both media subjected to analysis after preparation, values for F/2 and Daigo IMK calculated from their respective recipes, liquid fertilizer diluted tenfold with pure water and centrifuged at 6500×*g* for 10 min to precipitate suspended materials before analysis, and nitrite presence somewhat disturbed nitrate determinations in sewage water (indicated by parentheses)

### Use of liquid fertilizer from cattle feces as a nutrient source

Because sewage water was effective for *C. gracilis* cultivation, liquid fertilizer obtained as a waste material after methane fermentation of cattle feces was substituted in place of sewage water (Table 7). The liquid fertilizer contained high concentrations of phosphate and ammonium but no nitrate (Table 6). The 10% fertilizer (v/v) was centrifuged to remove suspended particles and one-quarter of the supernatant volume added to seawater (final concentration, 2% by vol). The resulting phosphate and ammonium concentrations were 4.0 and 60 ppm, respectively, which were roughly comparable with those in F/2 medium. Growth in this medium was reduced compared with that in F/2-enriched seawater (Table 7), but TAG accumulation was comparable in the same medium. When cells were subcultured a second time in the same culture medium, growth rate and cell density recovered to those observed in F/2 medium.

**Table 7:** Use of liquid fertilizer as the nutrient source in culture media

	2% supernatant of liquid fertilizer		0.5% liquid fertilizer		1% liquid fertilizer		2% liquid fertilizer	
	1st subculture	2nd subculture	1st subculture	2nd subculture	1st subculture	2nd subculture	1st subculture	2nd subculture
Doubling time (h)	17.6 ± 0.5	12.5 ± 0.3	10.7 ± 0.1	14.5 ± 0.1	11.8 ± 0.1	11.6 ± 0.3	15.9 ± 0.7	15.8 ± 0.5
Cell density on 4th day (10^6^ cells/mL)	3.69 ± 0.06	4.87 ± 0.10	4.73 ± 0.12	4.61 ± 0.06	5.19 ± 0.08	6.65 ± 0.16	6.12 ± 0.09	6.88 ± 0.11
Cell density on 10th day (10^6^ cells/mL)	6.06 ± 0.37	6.69 ± 0.27	5.38 ± 0.05	5.59 ± 0.10	7.71 ± 0.01	8.75 ± 0.08	11.6 ± 0.1	9.88 ± 0.05
TAG on 10th day (mg/L)	220 ± 5	181 ± 3	94.9 ± 3.4	93.1 ± 3.8	165 ± 4	217 ± 15	213 ± 2	270 ± 15

Liquid fertilizer diluted tenfold (10% by vol) with pure water; resulting 10% solution, with or without removing suspended materials, diluted further to 5, 10, and 20% (v/v) with seawater to obtain indicated amounts of liquid fertilizer culture media; cells cultured in resulting medium; cells used to inoculate liquid fertilizer medium cultivated in F/2-enriched seawater (1st subculture) or subcultured once in same liquid fertilizer medium (2nd subculture); and *n* = 3

Liquid fertilizer was also tested without removing suspended particles. In medium with 0.5% liquid fertilizer (by vol), *C. gracilis* cells grew at a rate comparable to that observed in F/2-enriched seawater, but TAG production was reduced by about half. When cells were subcultured a second time in the same medium, growth was slightly reduced. However, when cells were cultivated with relatively high fertilizer concentrations (1 or 2% by vol), TAG production was not reduced and growth rate did not decrease after a second subculture in the same medium.

### Fatty acid composition under various culture conditions

**Fig. 3:**
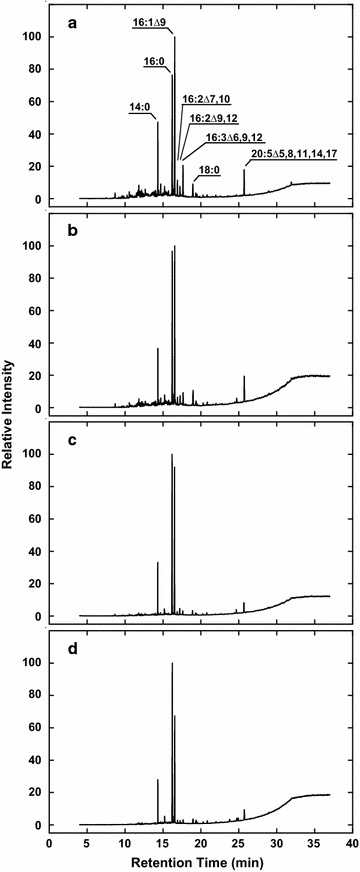
GC–MS chromatograms of FAME derived from *C. gracilis* cells grown in F/2-enriched seawater without vitamins. Cells harvested at day 2, 4, 7, and 10 (**a**–**d**, respectively) after culture start; extracted lipids methyl-esterified and subjected to GC–MS; and peak identities indicated on **a**

**Fig. 4:**
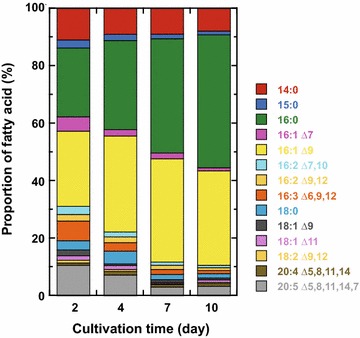
Changes in FA composition of *C. gracilis* cells during the course of growth determined by GC–MS in Fig. 5. *C. gracilis* grown in F/2-enriched seawater without vitamins, cells harvested at indicated times, identities of *colored boxes* indicated at right, each FA shown as a proportion of TFA, and mean values plotted (*n* = 3)

**Fig. 5:**
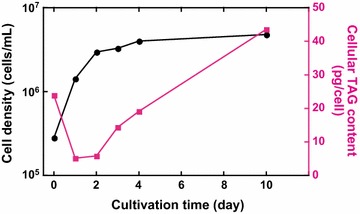
Changes in cellular TAG content during time course of growth. *C. gracilis* grown in F/2-enriched seawater without vitamins, and TAG content and cell density determined at indicated times

The *C. gracilis* fatty acid (FA) composition, assessed as FA methyl esters (FAME) by gas chromatography–mass spectroscopy (GC–MS, Fig. 3), changed during the time course of growth (Fig. 4). When cells were grown in F/2-enriched seawater, palmitic and palmitoleic acids (16:0 and 16:1, respectively) were the dominant fatty acids (24 and 26% of total FA, TFA, respectively) on the 2nd day of culture. Myristic acid and EPA (14:0 and 20:5 at 11 and 10% of TFA, respectively) were also remarkable. During the course of growth, 16:0 and 16:1 proportions increased such that their sum reached ~80% of TFA on the 10th day of culture. Conversely, the proportions of other FAs, including 14:0 and 20:5, decreased. DHA (22:6) was barely detected using the present method of extraction and analysis. Changes in FA composition appeared to be correlated with changes in TAG production in these cells (Fig. 5).

**Fig. 6:**
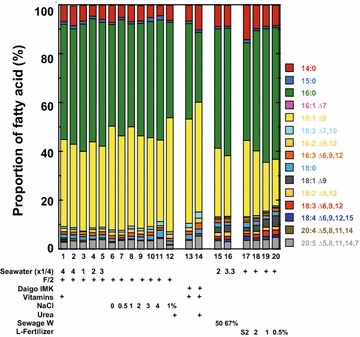
FA composition of *C. gracilis* cells grown in various culture media. Cells grown for 10 days in various culture media, including media with low salinity; FA composition assessed by GC–MS, *colored box* identities on right; each FA as proportion of total FA; and mean values plotted (*n* = 3). The following media used F/2-enriched seawater with vitamins or without vitamins (*1* and *2*, respectively); F/2-enriched seawater with 1, 2, or 3% sea salts (*3–5*, respectively); F/2-enriched NaCl medium with 0, 0.5, 1, 2, 3, or 4% NaCl (*6*–*11*, respectively); F/2-enriched 1% NaCl medium with urea (*12*); Daigo IMK medium (*13*); Daigo IMK medium with urea (*14*); seawater diluted with one or two volumes of sewage water (*15* and *16*, respectively); 2% liquid fertilizer supernatant in seawater (*17*); 2, 1, or 0.5% liquid fertilizer (*18–20*, respectively) in seawater; and vitamins added to media *1*, *13*, and *14*

Figure 6 shows FA compositions of cells grown under various culture conditions on the 10th day of culture. When cells were cultivated in F/2-enriched seawater, the two most abundant FAs were 16:0 and 16:1 (47 and 36% of TFA, respectively), which accounted for over 80% of TFA. Among the minor FAs, 14:0 and EPA (6.8 and 2.8% of TFA, respectively) were remarkable. Although the proportion of 16:1 was smaller when liquid fertilizer was used as a nutrient source (Fig. 6, samples 17–20), in general, the sum of 16:0 and 16:1 exceeded 70% of TFA under all conditions.

### Large-scale *C. gracilis* cultivation in open air

**Fig. 7:**
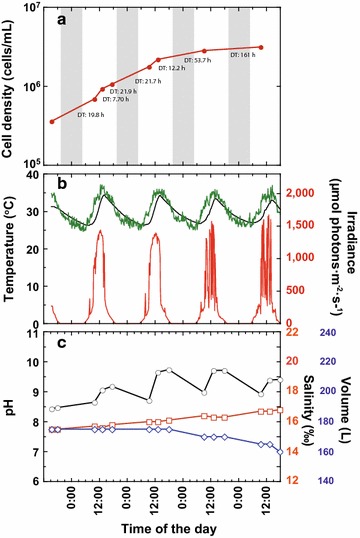
Open-air large-scale cultivation of *C. gracilis*. **a** Changes in cell density. DT designates doubling time between two consecutive time points. **b** Changes in ambient air, water temperatures, and irradiance (*green*, *black*, and *red lines*, respectively). **c** Changes in pH, salinity, and volume of culture medium (*black*, *red* and *blue lines*, respectively). Experiment performed from August 3 to 7, 2015

To examine scalability for industrial purposes, a large-scale cultivation of *C. gracilis* was performed in open air without any overhead shelter, using ~200 L of 1% NaCl-based medium supplemented with *f* [78] (Fig. 7). Ambient air and water temperatures fluctuated with the time of day with a mean value of ~30 °C (Fig. 7b). The highest irradiance at the culture medium surface was about 1700 µmol photons m^−2^ s^−1^. Although the irradiance as well as temperature were extremely high compared with laboratory experiments, *C. gracilis* grew well, showing the highest DT at 7.70 h and an average DT of 17.8 h during the first 2 days (Fig. 7a), and cell density after 4 days at 3.14 × 10^6^ cells/mL. As there was no rain during the 4 experimental days, the culture volume decreased slightly and salinity increased slightly through evaporation (Fig. 7c). The pH increased in daytime and decreased at night and the volume-specific TAG was 15.8 mg/L and cell-specific TAG was 5.04 pg/cell after 4 days of culture.

### Pigment production under various culture conditions

Amounts of accumulated pigments, such as fucoxanthin and Chls, were determined after 4 and 8 days (96 and 192 h, respectively) of *C. gracilis* cultivation (Table 8). When cells were grown in F/2-enriched seawater under continuous light of 50 µmol photons m^−2^ s^−1^, Chl *a* concentration reached 3.38 mg/L after 4 days of culture. Amounts of Chl *c*_1+2_ and fucoxanthin, by weight, were ~20 and 60%, respectively, relative to Chl *a* at the same time point. Over the next 4 days of culture, the Chl *a* concentration decreased by 12%, to 2.96 mg/L, while cell density increased 1.6-fold (Fig. 1). After 8 days of culture, the concentrations of fucoxanthin and Chl *c*_1+2_ also decreased by 12% compared with those after 4 d of culture. When cells were grown in Daigo IMK medium, pigment production was similar to that of cells grown in F/2 medium after 4 d of culture. Between 4 and 8 days of culture, the trend was slightly modified, with Chl *a* and *c*_1+2_ concentrations decreased to a small extent and fucoxanthin increased by 9%. Chl *c*_3_ and nonpolar Chl *c* were not detected here.

**Table 8:** Pigments in *Chaetoceros gracilis* cells grown for 4 or 8 days in F/2 or Daigo IMK medium

Pigments	Amount of pigments (mg/L)			
	F/2		Daigo IMK	
	4 days	8 days	4 days	8 days
Chlorophyll *a*	3.38 ± 0.39	2.96 ± 0.07	3.49 ± 0.06	3.36 ± 0.07
Chlorophyll *c*	0.734 ± 0.070	0.646 ± 0.018	0.761 ± 0.008	0.741 ± 0.013
Fucoxanthin	1.95 ± 0.31	1.71 ± 0.05	2.02 ± 0.08	2.20 ± 0.06
Diadinoxanthin	0.524 ± 0.030	0.611 ± 0.030	0.513 ± 0.023	0.597 ± 0.009
ß-Carotene	0.0962 ± 0.0113	0.0696 ± 0.0042	0.0957 ± 0.0032	0.0720 ± 0.0055

*Chaetoceros gracilis* cells cultivated in F/2-enriched seawater or Daigo IMK culture medium, pigments quantified on days 4 and 8, and *n*

Because various culture conditions were effective for growth and TAG accumulation, pigment production was assessed using urea, sewage water, or liquid fertilizer as a nitrogen source (Table 9). When urea was used instead of nitrate, the pigment production trend was similar to that with nitrate. As sewage water collected after a rain was used for this experiment, phosphate and ammonium concentrations in the working culture medium of 67% sewage water were low, at 1.3 and 6.7 ppm, respectively. Under such low nutrient availability, *C. gracilis* grew at reduced growth rate, reaching 2.32 ± 0.49 × 10^6^ cells/mL after 4 days of culture. Because of this reduced growth, the pigments produced were low. During prolonged storage of diluted liquid fertilizer, for >1 year, phosphate and nitrate concentrations in 2% (v/v) liquid fertilizer culture medium were decreased by about half. With this culture medium, *C. gracilis* grew at a somewhat lower rate compared with F/2 medium (4.02 ± 0.30 × 10^6^ cells/mL after 4 days of culture) and the amount of accumulated pigments reduced to about three-quarters of F/2 medium.

**Table 9:** Pigment contents in *Chaetoceros gracilis* cells grown in various culture media

Pigments	Amount of pigments (mg/L)							
	F/2		Urea-F/2		67% sewage water		2% liquid fertilizer	
	96 h	240 h	96 h	240 h	96 h	240 h	96 h	240 h
Chlorophyll *a*	3.77 ± 0.22	3.22 ± 0.17	3.45 ± 0.16	3.00 ± 0.09	0.778 ± 0.106	0.710 ± 0.043	2.85 ± 0.24	2.33 ± 0.04
Chlorophyll *c*	0.778 ± 0.050	0.685 ± 0.040	0.672 ± 0.028	0.614 ± 0.014	0.163 ± 0.018	0.136 ± 0.010	0.549 ± 0.036	0.399 ± 0.006
Fucoxanthin	2.01 ± 0.02	1.99 ± 0.10	1.95 ± 0.16	1.89 ± 0.05	0.433 ± 0.063	0.444 ± 0.029	1.39 ± 0.01	1.22 ± 0.05
Diadinoxanthin	0.535 ± 0.059	0.564 ± 0.007	0.493 ± 0.020	0.529 ± 0.018	0.140 ± 0.019	0.193 ± 0.013	0.361 ± 0.071	0.375 ± 0.011
ß-Carotene	0.115 ± 0.004	0.0759 ± 0.0025	0.0681 ± 0.0310	0.0695 ± 0.0022	0.0237 ± 0.0045	0.0255 ± 0.0010	0.0841 ± 0.0033	0.0670 ± 0.0006
Phosphate (ppm)	3.46		3.46		1.3		1.73	
Nitrate (ppm)	45		0		0		0	
Ammonium (ppm)	0		0		6.7		33	
Cell density on 4th day (10^6^ cells/mL)	4.25 ± 0.37		4.90 ± 0.31		2.32 ± 0.49		4.02 ± 0.30	
Cell density on 10th day (10^6^ cells/mL)	6.19 ± 0.1		6.43 ± 0.47		4.05 ± 0.41		4.30 ± 0.22	

*Chaetoceros gracilis* cultured in F/2-enriched seawater inoculated in F/2-enriched seawater, Urea-F/2 medium contained urea not nitrate at same molar concentration, seawater mixed with two volumes of sewage water (67% sewage water medium) or seawater mixed with one-fifth of 10% (v/v) liquid fertilizer (2% liquid fertilizer medium), pigments quantitated on days 4 and 10, and *n* = 3

Different from large-scale cultivation performed here in the open air at ~30 °C, all laboratory cultivations in this work were performed at 20 °C under continuous light. Thus, pigment production was assessed by mimicking open-air conditions, with a photoperiod of 12/12 h, light/dark, with 50 and 100 µmol photons m^−2^ s^−1^ at 30 °C (Table 10). When cells were grown under 50 µmol photons m^−2^ s^−1^ for 4 days, the amount of Chl *a* (5.09 mg/L) was larger by ~50% compared with the value for the cells grown for 4 days under continuous light shown in Table 8 (3.38 mg/L). After a further 6 d of culture, Chl *a* amounts increased by ~17% (5.98 mg/L), which was different from the trend shown in Tables 8 and 9, in which Chl *a* amounts decreased. Similar trends in pigment production were observed for other pigments. When cells were grown under higher irradiance for 4 days, the amounts of Chls *a* and *c*_1+2_ and fucoxanthin were smaller than in cells grown under lower irradiance but still larger than those in cells grown at lower temperature and continuous light. After an additional 6 days of cultivation, these pigments slightly decreased. Only diadinoxanthin increased under higher irradiance, which might have reflected diadinoxanthin cycle activation to protect cell photosystems from high light.

**Table 10:** Amounts of pigments in *Chaetoceros gracilis* cells grown under 12 h light/12 h dark at 30 °C

Pigments	Amount of pigments (mg/L)			
	50 µmol photons m^−2^ s^−1^		100 µmol photons m^−2^ s^−1^	
	96 h	240 h	96 h	240 h
Chlorophyll *a*	5.09 ± 0.15	5.98 ± 0.65	4.55 ± 0.10	4.34 ± 0.09
Chlorophyll *c*	0.965 ± 0.031	0.924 ± 0.028	0.844 ± 0.023	0.771 ± 0.020
Fucoxanthin	2.67 ± 0.11	2.92 ± 0.09	2.52 ± 0.02	2.45 ± 0.08
Diadinoxanthin	0.575 ± 0.034	1.18 ± 0.05	0.853 ± 0.034	0.962 ± 0.013
ß-Carotene	0.186 ± 0.006	0.203 ± 0.011	0.180 ± 0.004	0.164 ± 0.003
Cell density (10^6^ cells/mL)	4.54 ± 0.13	6.45 ± 0.27	5.39 ± 0.07	6.92 ± 0.16

Cells cultivated in F/2-enriched seawater under a photoperiod of 12 h light/12 h dark with 50 or 100 µmol photons m^−2^ s^−1^ at 30 °C, pigments quantitated on days 4 and 10, and *n* = 3

## Discussion

Diatoms are known as highly productive algae that accumulate high amounts of neutral lipids in their cell bodies [8]. The advantages of diatoms over other microalgae for production applications have been fully discussed elsewhere [11, 79]. Among the many diatom species, the superiority of *C. gracilis* was demonstrated in this study.

### Vitamins are not essential for growth

Cost is an important consideration for industrial production of valuable metabolites or biofuels using phytoplankton. Vitamins, especially vitamin B_12_, are included in many culture media [80], and many microalgae require vitamins to grow in culture [81]. However, there are also eukaryotic microalgae that do not need vitamins in culture, such as the primitive red alga *Cyanidioschyzon merolae* [82, 83]. For large-scale phytoplankton culture, the vitamin costs can be considerable. In this work, the effects of vitamins on growth and TAG production in *C. gracilis* were evaluated by comparison with a model diatom, *P. tricornutum* (Fig. 1; Table 1). The elimination of vitamins slightly decreased *P. tricornutum* growth and reduced TAG accumulation to one-third of normal using culture medium containing vitamins. It has been reported that vitamin B_12_ highly accelerates growth of a marine centric diatom, *T. pseudonana* [81]. Interestingly, neither growth rate nor TAG content of *C. gracilis* were affected by vitamin elimination; moreover, both growth and TAG accumulation here were superior in *C. gracilis*, compared with many other phytoplankton, including other diatoms [9]. Although there remains a possibility of contributions by coexisting microbes, *C. gracilis* was cultivated here without expensive vitamins, which would substantially reduce cultivation costs.

### Tolerance to environmental stress

Diatom growth can be affected by many environmental variables, including temperature, salinity, and nutrient concentrations. *C. gracilis* grows well under a wide range of temperatures, including 15–35 °C [84] and also grows well under a wide range of light intensities [34]. In the present study, *C. gracilis* was shown to grow well in media with various sea salt and NaCl concentrations (Tables 3, 4, respectively). In the field, culture media salinity increased on sunny days with high temperatures and decreased on rainy days. Although *C. gracilis* is a marine diatom, it can acclimate to environments with a wide range of salinity, such that it grew well and accumulated large amounts of TAG under almost all salinity conditions tested in this study (0–3% NaCl). *C. gracilis*’ ability to tolerate substantial variations in temperature, light intensity, and salinity would both facilitate industrial-scale cultivation of this diatom and reduce costs associated with controlling culture conditions. The best media found here, fertilizer + seawater, should be further tested against a range of realistic growth temperatures, as all laboratory cultivations were performed at 20 °C. *P. tricornutum* also tolerated various salinities and, given that *P. tricornutum* was isolated from brackish water, low salinity might not be a problem for this diatom. Nonetheless, growth rate and TAG accumulation were much higher in *C. gracilis* than in *P. tricornutum*.

### Use of sewage water and liquid fertilizer for cultivation

As *C. gracilis* was found to grow in media with low salinity, the use of sewage water and liquid fertilizer was investigated for cultivating this diatom. Mixing seawater with sewage water or liquid fertilizer substantially reduced media salinities. In both sewage water and liquid fertilizer, nitrate concentrations were low or negligible and thus ammonium the dominant nitrogen source (Table 7). Nevertheless, by consuming only nutrients contained in sewage water or liquid fertilizer, *C. gracilis* grew well and accumulated large amounts of TAG (Tables 6, 8). In coastal areas, there are usually many sewers that gather wastewater and, thus, establishing cultivation systems near sewage treatment plants could drastically reduce cultivation costs. Furthermore, large amounts of carbon dioxide released from activated sludge in these plants could support active photosynthesis in such cultivation system.

Large quantities of liquid fertilizer are produced as a waste product of methane fermentation of cattle feces, leading to the desire for new uses for this waste product. *C. gracilis*’ growth characteristics were better in media with liquid fertilizer than in media with sewage water (Tables 6, 8). The use of such supplements to cultivate this diatom could reduce cultivation costs and contribute to waste management.

Before expanding these culture media to large-scale or long-term cultivation, several points should be addressed. The use of liquid fertilizer supernatant might increase the cost by requiring suspended material removal. The use of liquid fertilizer without removing suspended materials would be more reasonable. Here, the use of 0.5% liquid fertilizer culture media reduced growth and TAG production at 2nd subculture. Second subculture with sewage water culture media also decreased growth and TAG production (Table 6). In these cases, culture media of precultures should be selected to support reasonable growth in large-scale culture. However, appropriate liquid fertilizer concentrations yielded reasonable growth rates and TAG production even in the 2nd subculture (Table 8), which would contribute in repeated cultivations and/or long-term cultivation. The use of 2% liquid fertilizer decreased the growth rate but increased TAG production in the 2nd subculture. This might have resulted from reduced light intensity caused by suspended materials in the liquid fertilizer, as the ODs of 2% liquid fertilizer at 675 and 730 nm were as high as 1.124, and 1.008, respectively.

### FA composition

Microalgae are expected to become a future source of long-chain *n*-3 polyunsaturated FAs, such as EPA and DHA [85], which are valuable materials because they are clinically important [24] and currently obtained mainly from fish oils. In this study of *C. gracilis*, DHA was scarcely detected, but EPA was the third or fourth most abundant FA (Figs. 4, 5). The EPA proportion in TFA decreased during the time course of growth, which was caused by increased cellular TAG production. However, total cell number also increased, resulting in increased EPA yield.

Although the FA composition changed somewhat during the course of growth, two FAs (16:0 and 16:1) accounted for over 70% of TFA throughout the growth period (Fig. 4). Furthermore, after 10 days of cultivation, FA composition was not affected by the salinity or nutrient conditions tested here. Therefore, *C. gracilis* was considered to be a stable FA source for biodiesel production.

### Large-scale cultivation of *C. gracilis* in open air

*Chaetoceros gracilis* grew well in large-scale cultivation in the open air without CO_2_ enrichment. The highest and average DT during the first 2 days were 7.7 and 17.8 h, respectively (Fig. 7a). The average growth rate was also comparable with laboratory experiments tested with *C. gracilis* using similar a light/dark cycle at 28 °C (DT of ~12 h, [86]). It was also comparable to the growth rate of *C. muelleri* tested at 30 °C under continuous light (DT of ~12 h, [84]). In addition, this was comparable with the highest growth rate obtained with a similar light/dark cycle in small-scale laboratory culture at 20 °C (DT of 15 h for *Cylindrotheca fusiformis* and *Pseudo*-*nitzschia pseudodelicatissima* [9]). Although pigment analysis using cells cultivated in open-air was not performed, high production of valuable pigments, such as fucoxanthin, could be achieved with *C. gracilis*, as this species showed high pigment production under an environment simulating open-air cultivation (Table 10). Because cells grew well under open-air light conditions (Fig. 7), light intensity at the water surface, much higher than that used in Table 10, should not have affected pigment production negatively.

### Pigments

Chl *a* is one of the dominant diatomaceous photosynthetic pigments and is currently purified from microalgae or spinach. Industrial diatom cultivation in the near future could yield large amounts of Chl *a* as a cellular debris residue from other processes. Chls have several potential uses, such as serving as sensitizers for light-capturing materials in solar panels.

Chl *c*, having no phytol group, is a polar molecule compared to other Chls, a feature that increases the variety of its potential uses. Allophycocyanin, a water-soluble blue pigment and photosynthetic chromophore in cyanobacteria and red algae, is used as a fluorescent label for immunodetection and as a food colorant [87]. Chl *c*, a brown-yellow pigment, can also be used as a food colorant [88]. Furthermore, Chl *c* is a porphyrin, similar to the heme in hemoglobin, whereas other types of Chl are chlorins. This structural similarity with heme could allow Chl *c* to be used for pharmaceutical purposes.

Fucoxanthin, one of the xanthophylls expected to be commercially produced [89], could be beneficial to human health. There are many reports of fucoxanthin’s inhibitory effects on cancer cells [28–33], and its antioxidant effects are also well known [37]. Fucoxanthin is usually purified from edible brown algae, such as *Laminaria* spp. [37], which are abundant and useful as human food. Diatoms can be ideal biofactories for industrial fucoxanthin production because they use solar energy to drive biosynthesis. It is noteworthy that both *C. gracilis*’ fucoxanthin content (Table 8) and growth rates were high in the present study. Furthermore, nutrient costs could be kept low by using wastewater as a nutrient source (Tables 5, 7).

For fucoxanthin purification from brown algae, 1 g of wet algal body, for example, is soaked in 30 mL of methanol for 2 days, producing a fucoxanthin yield of 0.24–0.43 mg/g, as has been reported by Mori et al. [37]. Comparable fucoxanthin amounts (~0.3 mg fucoxanthin/g) from 1 g of wet brown algal body could be purified here from about 150 mL of culture at stationary phase (Table 8). In accordance with the usual wet weight of this diatom, at 0.5–0.6% (w/v) of culture medium, after 4–10 days of cultivation using F/2 medium in the present experiments, the wet weight here should be ~0.9 g for ~150 mL of culture. If methanol is used in the same proportion to diatom wet weight as for brown algae, comparable methanol amounts are needed. However, as diatoms are unicellular organisms, the required solvent should be less than that calculated above and the extraction time for pigments extremely short, at <1 h. Therefore, fucoxanthin could be purified, with little oxidation damage, via a short isolation period and with lower costs compared with current fucoxanthin sources.

The costs for pigment production also depend on cultivation methods. Here, urea was an effective nitrogen source for growth (Table 5), which reduced the cultivation cost by taking the place of nitrate. With this replacement, pigment production comparable to F/2-enriched culture medium was achieved (Table 9). Sewage water and liquid fertilizer were effective low-cost additives as main nitrogen and phosphate sources to support growth and TAG accumulation (Tables 5, 7). Because sewage water used to cultivate cells for pigment analysis was collected just after a rain, phosphate and nitrogen source concentrations were low. Concentrations of such nutrients in diluted liquid fertilizer were also decreased after long-term storage. These effects on pigment production when using sewage water and liquid fertilizer were not considered large after examination of the results shown in Tables 5 and 7. However, limited levels of pigment production were supported even under such poor conditions.

## Conclusions

In this report, a marine centric diatom, *C. gracilis*, was shown to possess high tolerances to environmental stressors, such as changes in salinity, and to grow well under a variety of conditions, yielding large amounts of TAG and EPA. Cell growth and TAG production by *C. gracilis* were superior to those of *P. tricornutum*, which has been used in many studies into its potential industrial applications, including oil production [64, 90]. Furthermore, *C. gracilis* growth was faster than many other diatoms and green algae and, although direct comparisons are difficult, lipid production was also higher in *C. gracilis* than in these organisms [9]. While growth and TAG production of *C. gracilis* were satisfactory in most culture media tested, F/2-enriched with 1% NaCl solution was the most effective for both growth and TAG accumulation. Under this condition, *C. gracilis* showed clear advantage over other microalgae including oleaginous diatoms on the growth rate and TAG productivity (Table 11). It is also noteworthy that only two fatty acids are dominated FAME compounds; palmitic (16:0) and palmitoleic (16:1) acids in *C. gracilis*. Twenty percent liquid fertilizer was also effective, supporting high TAG production at a reasonable growth rate.

**Table 11:** Comparison of growth, lipid production, and fatty acid composition between oleaginous microalgae

	Doubling time (h)	Amount of TAG or FAME (mg/L)^a^	TAG productivity (mg/L per day)	Lipid or FAMA productivity (mg/L per day)	Percentage of TAG in lipid	Fatty acid composition (% w/w) of total FAMEs											References
						14:0	16:0	16:1	16:2	16:3	16:4	18:0	18:1	18:2	18:3	20:5	
Diatom																	
*Chaetoceros gracilis*	9.2	272 (TAG)	27.2			6.7	42.1	40.8	2.7	1.5		1.1				3.9	This work (1% NaCl)
*Phaeodactylum tricornutum*	14.6	183 (TAG)	18.3			4.9	38.0	42.5	0.5	1.1		1.4	4.8			6.7	This work (1% NaCl)
*Navicula* JPCC DA0580	11.3^b^			26.4			21.4^c^	25.1^c^				0.3^c^	0.5^c^	0.3^c^			[94]
*Thalassiosira weissflogii* P09	16		3.7^d^	7.3	51.0												[9]
*Thalassiosira weissflogii* CCMP1010	22		2.6^d^	4.9	53.0												[9]
*Cylindrotheca fusiformis* CCMP 343	15		0.9^d^	4.8	18.0												[9]
*Nitzschia* CP2a	74	39				6.0	30.1	31.6	2.8	0.0	0.0	5.7	2.0	0.8	1.8	12.5	[95]
*Nitzschia* CP3a	96	51				5.3	28.8	27.3	3.3	0.0	0.0	5.3	2.9	1.3	1.2	14.5	[95]
*Cymbella* CP2b	31	41				9.6	37.7	38.3	1.6	0.0	0.0	6.6	1.2	0.3	0.1	3.9	[95]
*Cylindrotheca closterium* Sl1c	31	35				0.6	0.2	4.7	9.0	23.3	1.8	35.2	10.1	0.2	10.8	0.4	[95]
*Tetraselmis* M8	38	48				0.9	28.3	1.8	14.6	18.3	0.3	19.8	8.1	0.2	3.4	0.3	[95]
Cyanobacterium																	
*Spirulina maxima*				8.6^e^		0.3	40.2	9.2		0.4	0.2	1.2	5.4	17.9	18.3		[96]
Eustigmatophyceae																	
*Nannochloropsis salina* CCMP369	44		1.5^d^	7.3	20.0												[9]
*Nannochloropsis* sp.				25.8^e^		7.2	23.4	26.9	0.4	0.5		0.5	13.2	1.2		14.3	[96]
Grren algae																	
*Neochloris oleabundans*				26.1^e^		0.4	19.4	1.9	1.7	1.0	7.2	1.0	20.3	13.0	17.4		[96]
*Chlorella* NKG400014	12.7^b^			13.1			6.7^c^	0.3^c^				1.1^c^	9.1^c^	2.4^c^	15.5^c^		[94]
*Chlorella vulgaris*				9.2^e^		3.1	25.1	5.3		1.3	4.1	0.6	12.6	7.2	19.1	0.5	[96]
*Chlorella* BR2	37	9				1.7	31.7	4.7	1.4	8.8	0.04	29.3	11.9	6.1	0.4	0.1	[95]
*Chlorella vulgaris*				4													[97]
*Scenedesmus obliquus*				15.9^e^		1.5	21.8	6.0	4.0	0.7	0.4	0.5	17.9	21.7	3.8		[96]
*Dunaliella tertiolecta*				20.0^e^		0.5	17.7	0.9	3.0	1.2	10.6		4.9	12.4	30.2		[96]
*Dunaliella tertiolecta*	36	18				0.5	19.9	0.4	1.4	4.2	23.3	16.7	0.8	1.9	30.0	0.1	[95]
*Dunaliella salina* CCAP19/18	83		0.4^d^	5.2	7.0												[9]
*Dunaliella tertiolecta* CCMP 1320	51		1.7^d^	9.7	17.0												[9]
*Chlamydomonas* CCMP222	30		0.2^d^	3.0	7.0												[9]
Higher plants																	
Canola oil							4.6	0.2				2.1	64.3	20.2	7.6		[98]
Palm oil						1.1	41.9	0.2				4.6	41.2	10.3	0.1		[98]
Soybean oil							10.5					4.1	22.5	53.6	7.7		[98]

Fatty acid composition of typical oils from higher plants are also presented

All data of microalgae were selected among those grown with air (without enriching CO_2_)

^a^Values at the end of culture test

^b^Calculated from the data on Fig. 1 in [94]

^c^Values of % toward dry cell weight

^d^Calculated from the data on Table 1 in [9]

^e^Calculated from the data on Table 3 in [96]

It was also shown that *C. gracilis* produced high amounts of TAG without the need for nitrogen or silica deprivation, which is frequently imposed on many other microalgae to induce lipid production [17, 23]. Processes for producing nitrogen or silica deprivation require cell transfer into nutrient-free medium, which incurs added labor, high costs, and complicates scalability. The ability of this microalga to accumulate TAG without nutrient deprivation is important for low-cost scalability.

A stable and efficient genetic transformation technique for *C. gracilis* was developed in a previous study by this group [74]. Using this highly productive diatom, a platform to produce valuable metabolites could be constructed by introducing exogenous genes. Because *C. gracilis* produces large amounts of fucoxanthin and lipids, derivatives of these molecules could also be produced in large quantities. In addition, *C. gracilis* grows well and produces abundant fucoxanthin and lipids using sewage water or liquid fertilizer derived from cattle feces without carbon dioxide supplementation. Thus, production costs could be quite low. *C. gracilis* shows great potential as a biofactory.

## Methods

### Diatom strains and growth conditions

A centric marine diatom, *Chaetoceros gracilis* Schütt (UTEX LB 2658), and a pennate marine diatom, *Phaeodactylum tricornutum* Böhlin (UTEX 642), were used in this study. The cells were grown photoautotrophically in artificial seawater (sea salts; Sigma-Aldrich, Inc., St. Louis, MO, USA, or Marine Art SF-1; Tomita Pharmaceutical Co., Ltd., Naruto, Japan) supplemented with F/2 (half-strength medium *f* [78]) or Daigo IMK medium (Nihon Pharmaceutical Industry Co., Ltd., Tokyo, Japan) at 20 °C and with ambient air bubbled through the media [50]. The concentrations of copper and molybdenum were 10 times that of original F/2 without any positive/negative effects on growth. The initial culture volume was 70 mL in glass tubes (30 mm dia × 200 mm long; ~90 mL vol). Because sea salts and Marine Art SF-1 did not contain silicate, 15 mg/L NaSiO_3_·9H_2_O was added to all culture media. When necessary, vitamins (0.5 µg/L B_12_, 0.5 µg/L biotin, and 100 µg/L thiamine-HCl) were added to the medium. Where indicated, a NaCl-based solution, containing variable concentrations of NaCl, 1.33% MgSO_4_·7H_2_O, 0.147% CaCl_2_·2H_2_O, 0.080% KCl, and 0.025% NaHCO_3_ (w/v) were used instead of artificial seawater. Urea as a nitrogen source was assessed by substituting urea for nitrate in media at the same molar concentration. All chemicals were of a high purity.

Sewage water and liquid fertilizer were used here as additives to culture media. Sewage water was obtained from Harima Kogen Higashi Waterworks, located near the Harima Campus for Science of the University of Hyogo, Hyogo Prefecture, Japan (35°55′N, 134°26′E). Liquid fertilizer, from cattle feces fermentation, was supplied by Marubeni Corp., Kyushu Branch (Fukuoka, Japan). To prepare working culture media by diluting seawater, trace metals and silicate were added to the same concentrations as in F/2 medium. All experiments were performed using cells subcultured at least twice in the same medium, unless otherwise indicated.

**Fig. 8:**
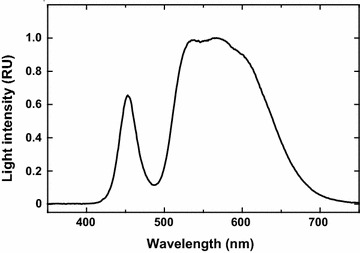
Spectral properties of LED light source used for *C. gracilis* culture. Wavelength-dependent intensity determined using a spectrometer BRC115 (B&W Tek, Inc., Newark, DE, USA) and normalized at peak wavelength

Continuous light was supplied by a warm, white, LED light source (VBL-SD150-LL; Valore Corp., Kyoto, Japan), whose spectral properties are shown in Fig. 8. The photon irradiance, measured using a quantum photometer (LI-COR Biosciences, Inc., Lincoln, NE, USA), was 50 µmol photons m^−2^ s^−1^. Cell density was determined by cell counting with a hemocytometer.

Open-air culture of *C. gracilis* was performed at the Harima Campus for Science of the University of Hyogo using a plastic tank (width/depth/height, 1045 × 785 × 802 mm, respectively) with ~200 L (~25 cm depth) of 1% NaCl-based culture medium supplemented with *f* [78]. Irradiance, illumination intensity, and temperatures were measured using a quantum photometer ML-020P, a pyranometer MS-602 (Eko Instruments Co., Ltd., Tokyo, Japan), and type T thermocouple probes, respectively, and data stored in a GL220 data logger (Graphtec Corp., Yokohama, Japan).

### Pigment analysis

Photosynthetic pigments were quantified using reversed-phase high-performance liquid chromatography by a LC-10AD with a SCL-10A system controller (Shimadzu Co., Ltd., Kyoto, Japan) and equipped with a Prodegy 5 column (ODS, 150 × 4.60 mm) supplied by Phenomenex Inc., (Torrance, CA, USA), as described previously [50], after extraction in *N,N*-dimethylformamide [35, 91]. Standard pigments were purchased from VKI Water Quality Institute (Hoersholm, Denmark). Chl *a* concentrations were determined as described by Porra et al. [92], using methanol as the solvent.

### Nutrient analyses

Concentrations of nutrients, including nitrate, nitrite, ammonium, phosphate, and silica, were determined using PACKTEST kits (Kyoritsu Chemical-Check Lab Corp., Tokyo, Japan), per manufacturer’s instructions. Obtained concentrations were 20% (20.3 ± 1.0% for nitrate and 20.8 ± 3.0% for phosphate, *n* = 2) lower than their expected values calculated from the F/2 recipe.

### TAG and FA analyses

Diatom cells were harvested from 2 mL of cell suspension by centrifugation at 1000×*g* and 4 °C. Lipids were extracted using the method of Bligh and Dyer [93] with slight modifications. In brief, the precipitated diatom cells were suspended in 1.2 mL of pure water and then 1.5 mL of chloroform and 3 mL of methanol were added. After cell disruption by ultrasonication for 5 min using a B2200 ultrasonic cleaner (Branson Ultrasonics Corp., Danbury, CT, USA), 1.5 mL of chloroform and 1.5 mL of pure water were added. After centrifugation at 400×*g* at 4 °C, the lipid-containing lower solvent phase was recovered. Additional lipids were recovered by the same procedure after adding 1.2 mL of chloroform to the upper aqueous phase from the previous extraction. The combined solvent phases were dried using a centrifugal concentrator (5305; Eppendorf AG, Hamburg, Germany) and then dissolved in 200 µL of 2-propanol. TAG contents were determined using a TAG quantification kit (Triglyceride E-Test Wako; Wako Pure Chemical Industries, Ltd., Osaka, Japan), based on a method using glycerol-3-phosphate oxidase and 3,5-dimethoxy-*N*-ethyl-*N*-(2′-hydroxy-3′-sulfopropyl)-aniline sodium salt, per manufacturer’s instructions. TAG concentrations were expressed as triolein equivalents.

After methyl esterification, the acyl composition in TAG was determined by GC–MS (GCMS-QP2010 Ultra, Shimadzu Corp., Kyoto, Japan). FA methyl esterification was performed with a FA methylation kit and the resulting FAMEs purified using a FAME purification kit (Nacalai Tesque, Inc., Kyoto, Japan). FAMEs were analyzed using an Agilent DB-23 column (30 m length, 0.25 mm diameter, and 0.25 mm film thickness; Agilent Technologies, Inc., Santa Clara, CA, USA). The inlet and detector temperatures were set to 250 °C, and 0.5–1.0 µL of each sample was analyzed using splitless injection and a constant flow rate of 1.22 mL/min. The oven temperature program was set to start at 50 °C for 2 min, increased to 180 °C at a 10 °C/min, held at 180 °C for 5 min, increased to 240 °C at 5 °C/min, and finally held at 240 °C for 5 min. FAMEs separated by GC were detected and analyzed on a mass spectrometer operating in scan mode. Mass spectra were recorded every 0.03 s over an *m/z* range of 60–600. FAMEs were identified using the NIST/EPA/NIH Mass Spectral Database, 2011 edition, and GC–MS solution software (Shimadzu Corp.).

## Abbreviations

Chl: chlorophyll
DHA: docosahexaenoic acid
DT: doubling time
EPA: eicosapentaenoic acid
FA: fatty acid
FAME: fatty acid methyl esters
GC–MS: gas chromatography–mass spectroscopy
TAG: triacylglycerol
TFA: total fatty acids
